# Assessment and Management of the Impact of Bovine Brucellosis on Dairy Herd Performance and Profitability

**DOI:** 10.3390/vetsci12121119

**Published:** 2025-11-25

**Authors:** Octavio Martínez-Guerrero, Pedro Hernández-Briano, Alberto Muro-Reyes, Yoana Tovar Maldonado, Adriana Lucía Perea-Lugo, Horacio Dávila-Ramos, Francisco J. Gutiérrez-Piña

**Affiliations:** 1 Unidad Académica de Medicina Veterinaria y Zootecnia, Universidad Autónoma de Zacatecas, General Enrique Estrada 98500, Zacatecas, Mexico; octavio.martinez@uaz.edu.mx (O.M.-G.); pedro.hernandez@uaz.edu.mx (P.H.-B.); amuro@uaz.edu.mx (A.M.-R.); yoana.tovar@uaz.edu.mx (Y.T.M.); adriana.perea@uaz.edu.mx (A.L.P.-L.); 2 Facultad de Medicina Veterinaria y Zootecnia, Universidad Autónoma de Sinaloa, Blvd. San Ángel s/n, Fraccionamiento San Benito, Culiacán 80246, Sinaloa, Mexico; ramos@uas.edu.mx

**Keywords:** *Brucella abortus*, *Holstein friesian*, Mexico, reproductive efficiency, milk yield, economic losses, endemic disease, biosecurity

## Abstract

**Background**: Bovine brucellosis is a high-impact zoonosis that threatens both dairy productivity and public health. Despite long-standing control programs in Mexico, evidence quantifying its combined productive, reproductive, and economic effects under intensive dairy systems remains limited. **Methods**: Farm records from two intensively managed *Holstein friesian* dairies operating under fully confined housing systems in Coahuila (Mexico) were analyzed to compare brucellosis-positive (BR) and brucellosis-negative (SIN) cows across five parities (*n* = 32,178 lactations; 15,507 BR; and 16,671 SIN). Milk yield, days open (DOs), services per conception (SPCs), days in milk (DIMs), abortion, metritis, retained placenta (RP), and culling to abattoir were evaluated using standard linear and generalized linear models. A financial assessment estimated losses associated with unproduced milk, extra DOs, extra inseminations, abortions, hormonal protocols, feeding during extra DOs, labor, and replacement costs. **Results**: BR cows consistently produced less milk (−3.5 to −5.0 L·cow^−1^·day^−1^) and exhibited longer DOs (+10 to +34 days) and higher SPCs (+0.4 to +1.0 in four of five parities; *p* < 0.05). Abortion incidence was higher in BR cows (1.74–2.79%) than in SIN cows (0.69–1.59%). Estimated total economic losses reached USD 21.84 M, including unproduced milk (USD 10.20 M), extended DOs (USD 1.36 M), feed cost during extra DOs (USD 0.68 M), additional services (USD 0.21 M), hormonal protocols (USD 0.10 M), labor (USD 0.06 M), abortions (USD 0.05 M), and excess replacements (USD 0.19 M). **Conclusions**: Under endemic conditions, brucellosis markedly reduces dairy herd productivity and reproductive performance. Strengthened sanitary control, enhanced biosecurity, strategic culling of positive animals, and structured reproductive programs represent the most cost-effective pathway to recover milk, shorten DOs, and minimize abortions.

## Simple Summary

Brucellosis is a contagious bacterial disease of cattle that can also infect people through direct contact with animals or the consumption of unpasteurized milk. In many Mexican dairy regions, infected cows are still present in herds, but there is little quantitative information on how much this disease reduces milk production and fertility or how much money it costs farmers. In this study, we analyzed farm records from more than thirty-two thousand periods of milk production after calving (lactations) of *Holstein friesian* cows kept in two large, fully housed dairies in northern Mexico. We compared cows that had tested positive for brucellosis with cows that repeatedly tested negative and were managed in a separate, healthy herd. Cows with brucellosis consistently produced several liters less milk per day, needed more inseminations to become pregnant, spent more time not pregnant between calvings, and had more abortions and other reproductive problems than healthy cows. Using these differences, we calculated that brucellosis caused large economic losses for the farms, most of them due to milk that was never produced, followed by the extra feed and labor required for cows that remained not pregnant for longer periods. Our findings show that living with brucellosis in dairy herds is costly and that investing in stricter control measures, such as better hygiene and animal movement control (biosecurity), systematic testing, and strategic culling of infected cows, can improve farm profitability and protect public health.

## 1. Introduction

Bovine brucellosis, caused by *Brucella* spp., is notable for its socioeconomic and public health impacts as a zoonosis, as well as for the productive and reproductive losses it causes in livestock systems [1]. In dairy herds, the disease causes abortions, retained placenta, increased services per conception, prolonged calving intervals, reduced milk production, and weak or low-birth-weight calves [2,3]. These losses are further compounded by high replacement costs for test-positive animals and trade restrictions [4,5]. In economic evaluations, the replacement cost is commonly expressed as the net replacement cost (i.e., heifer rearing cost minus salvage value) and should be evaluated jointly with milk losses and extended open days [6,7,8].

In cattle, infection is mainly due to *B. abortus* (~84.8%), less commonly *B. melitensis* (~15.2%) and rarely *B. suis* (~0–1%) [9,10]. Most infected animals abort once and may continue to shed the pathogen, facilitating transmission [11].

Mexico is an endemic country for brucellosis [12]. Disease control is governed by the National Campaign for the Control and Eradication of Brucellosis in Animals (NOM-041-ZOO-1995), which defines three phases: control, eradication, and free status [13]. Currently, approximately 57.4% of the country remains under the control phase, 29.6% is in eradication, and 12.9% is officially free of the disease, reflecting ongoing and persistent control challenges [13]. In 2003, national milk production totaled 13,104,852 thousand liters, valued at approximately MXN 102,048,080 thousand [14]. Mexico remains a net importer of dairy products, as domestic production does not fully meet national demand [15]. According to the regulation, animals that test positive must be culled [16], and limited financial incentives for replacement often lead producers to prioritize short-term profitability over long-term public health goals [12]. NOM-041 also permits the temporary management of positive cattle in controlled production units, allowing their milk to be utilized before culling [13].

Vaccination, testing, and culling decisions, as well as their combinations, should be evaluated through cost–benefit analyses to determine profitability at both the herd and policy levels [1,17]. However, comprehensive quantitative assessments of the combined productive, reproductive, and economic effects within Mexico’s intensive dairy systems are still lacking. Therefore, we evaluated the impact of bovine brucellosis on productivity, reproductive efficiency, and economic losses in *Holstein friesian* cows raised under intensive management in the dairy region of Coahuila, Mexico, considering herd performance indicators and management strategies aimed at mitigating disease effects.

## 2. Materials and Methods

### 2.1. The Herd and Area

This study involved *Holstein friesian* dairy cows from a commercial enterprise operating two fully housed dairies in the main dairy region of Coahuila, Mexico (25°48′36.4″ N, 103°13′37.7″ W), at an altitude of ~1120 m a.s.l., characterized by an arid–warm climate, an annual mean temperature ~22.3 °C, and annual precipitation ranging from 205 to 245 mm. Data were collected from March 2024 to January 2025.

Ethical review and approval were waived by the Institutional Bioethics and Animal Welfare Committee (Protocol # 2025/02/26) of the Unidad Académica de Medicina Veterinaria y Zootecnia at the Universidad Autónoma de Zacatecas (UAMVZ-UAZ), in accordance with the official Mexican norms for animal care (NOM-024-ZOO-1995; NOM-025-ZOO-1995). The study analyzed only anonymized, retrospective farm records without involving any handling or sampling of live animals.

The BR (brucellosis-positive) group included 2617 first-parity cows, 4705 second-, 4725 third-, 2926 fourth-, and 534 fifth-parity cows. The SIN (brucellosis-negative) group comprised 6414, 4473, 3782, 1562, and 440 cows, respectively, across the same parities. In total, 32,178 lactating cows were analyzed (15,507 BR; 16,671 SIN).

The dataset corresponded to a retrospective longitudinal cohort, where individual cows were followed across five consecutive lactations through herd management software. Records with incomplete or implausible reproductive or production data (e.g., missing calving dates, milk yield < 5 or >70 L/d) were excluded (<2% of total). Missing values within otherwise complete records were handled by listwise deletion to ensure analytical consistency.

### 2.2. Diagnosis and Management of Positive Animals

Since 2010, *B. abortus* strain RB51 vaccination has been implemented as part of the National Campaign for the Control and Eradication of Brucellosis in Animals (NOM-041-ZOO-1995) [13]. At the time of the last testing round (March 2024), the apparent prevalence of brucellosis was 9.2% (241/2617) among lactating cows tested in BR-designated barns. This estimate represents the proportion of animals that remained seropositive out of the total actively tested population during that round and not the cumulative proportion of all cows analyzed in this study.

Serological testing was performed annually, following the National Campaign for the Control and Eradication of Brucellosis in Animals (NOM-041-ZOO-1995) [13]. The card agglutination test was used for screening, and the rivanol precipitation test served as the confirmatory assay. Cows that tested positive on both the card and rivanol tests were considered infected and assigned to the BR group. No additional confirmatory assays (e.g., complement fixation or ELISA) were required, as the rivanol test is officially recognized as confirmatory under NOM-041-ZOO-1995.

Animals testing positive or suspect, according to campaign criteria, were segregated into one of two barns designated for BR cows and were excluded from subsequent serological testing rounds; these animals were not revaccinated with RB51. Brucellosis-negative animals (SIN group) tested negative on screening, were vaccinated annually with RB51, and were housed in a separate facility isolated from BR barns.

### 2.3. Feeding and Reproductive Management

All cows received the same total mixed ration (TMR) formulated to meet NEL, MP, RDP/RUP, and macro- and micromineral requirements according to production stage and physiological needs [18,19]. Feed was delivered twice daily.

Reproduction used a standard Ovsynch protocol (GnRH–7 d–PGF_2α_–2 d–GnRH–16 h AI); all artificial inseminations (AIs) were performed by trained technicians [20].

### 2.4. Replacements

Replacement was defined as culling to abattoir during lactation. For each parity and group (BR or SIN), the culling incidence rate was computed as (number of culling cases/total cows) × 100. Using the SIN group as the reference (counterfactual), the excess risk attributable to brucellosis was estimated using the risk difference (RD = risk_BR − risk_SIN), and 95% confidence intervals (CIs) were calculated using the Wald approximation for two independent proportions. Economic valuation was based on the net replacement cost, defined as the heifer rearing cost minus the salvage value [21]. The salvage value was parameterized at USD 57 per hundredweight (cwt) × 1547 lb (701.5 kg), equivalent to ≈USD 882 per cow (rounded to USD 900). The heifer rearing cost was set at USD 1500 under the base scenario, with sensitivity analyses conducted within realistic ranges. Accordingly, the net replacement cost was estimated at approximately USD 600 per culled cow [7,8,21].

### 2.5. Variables

From the farm database, the following variables were obtained: milk yield (L/d), days open (DOs, d), services per conception (SPCs, n), days in milk (DIMs, d), and abortions (%). Reproductive disorders included metritis, retained placenta (RP), and culling to abattoir (“Rastro”). In addition, age at conception and age at calving (both in months) were analyzed.

### 2.6. Economic Analysis

Economic losses were estimated as the sum of the following components: unproduced milk (USD) = (difference in daily milk yield between SIN–BR) × DIM × number of BR cows × milk price; days open (USD) = difference in DO × cost per open day (USD 5/d; sensitivity range USD 2.6–5.5) [22,23]; additional services (USD) = difference in SPC × cost per insemination (USD 250/event) [24,25]; hormonal protocols (USD) = number of treated cows × USD 12/cow [26]; feeding during extra DOs (USD) = additional DO × feeding cost per day (USD 2.5/d) [27]; labor (USD) = number of additional reproductive events × USD 7/event [27,28]; and replacements (USD) = excess replacements attributable to BR × net replacement cost per cow) [7,8,21]. The base milk price was USD 0.48/L (8.2 MXN/L) [29,30].

### 2.7. Statistical Analysis

Data were processed using SAS OnDemand for Academics (SAS® software, version 9.3; SAS Institute Inc., Cary, NC, USA) [31]. Milk yield was analyzed with PROC MIXED, including group status (BR vs. SIN) and parity as fixed effects and month as a random effect; when significant (*p* < 0.05), multiple comparisons were adjusted using Tukey’s test (α = 0.05). DOs, SPCs, and DIMs were analyzed using PROC GLM, with group status and parity as fixed effects. Reproductive disorders (abortion, metritis, RP, and culling) were evaluated using PROC GENMOD (binomial distribution, logit link), with group status and parity as fixed effects; for abortion analyses, only pregnant cows in the last third of gestation were included. Age at conception and age at calving were analyzed using PROC GLM, including group and month as fixed effects. Model diagnostics were performed to verify assumptions of normality, homoscedasticity, and independence.

Economic losses were calculated for each parameter, and sensitivity analyses were performed for milk price (USD 0.40–0.55/L) and cost per DOs (USD 2.6–5.5/d).

## 3. Results

### 3.1. Milk Yield, Reproduction, and Lactation Length

Average milk yield across parities was consistently lower (*p* < 0.05) in BR than in SIN cows. In parity 1, BR cows produced 29.6 L/d vs. 34.4 L/d in SIN cows (*p* < 0.0001); in parities 2 and 3, 32.4 vs. 36.3 L/d and 32.4 vs. 36.4 L/d (*p* < 0.0001); in parity 4, 32.4 vs. 36.9 L/d (*p* < 0.0001); and in parity 5, 34.4 vs. 38.4 L/d (*p* < 0.0001). This demonstrated a consistent reduction of ~3–5 L/cow/d in herds affected by brucellosis (Table 1).

DOs were significantly greater in BR than in SIN cows in all parities (*p* < 0.05): parity 1, 155 vs. 121 d (*p* < 0.0001); parity 2, 139 vs. 122 d (*p* < 0.0001); parity 3 vs. 125 d (*p* < 0.0001); parity 4, 136 vs. 121 d (*p* < 0.0001); and parity 5, 126 vs. 105 d (*p* < 0.0001).

SPCs were also higher (*p* < 0.05) in BR cows: parity 1, 3.5 vs. 2.5 (*p* < 0.0001); parity 2, 3.5 vs. 3.0 (*p* < 0.0001); parity 3, 3.4 vs. 3.0 (*p* < 0.0001); parity 4, 3.3 vs. 2.8 (*p* < 0.0001). In parity 5, SPCs did not differ significantly (2.4 vs. 2.5; *p* ˃ 0.05) (Table 1).

DIMs were consistently greater in BR cows than in SIN cows (*p* < 0.05), ranging 326–333 vs. 302–308 d in SIN cows for the first four parities (*p* < 0.0001); in parity 5, DIMs averaged 319 vs. 302 d (*p* < 0.0001).

### 3.2. Age at Conception and Calving

No group differences were found (*p* ˃ 0.05). Mean age at conception was 14.7 (BR) vs. 14.5 months (SIN), and age at calving was 23.7 vs. 23.5 months, respectively (Table 2).

### 3.3. Reproductive Disorders and Culling

Reproductive disorders were consistently more frequent in BR cows than in SIN cows (Table 3). The largest differences were observed for abortions, with rates of 2.42% and 1.95% in BR cows during parities 2 and 3, compared with 0.89% and 0.69% in SIN cows (*p* < 0.05). Metritis was diagnosed in 0.51% and 0.47% of BR cows, compared with 0.04% and 0.19% in SIN cows in parities 2 and 3, respectively. RP was likewise more frequent in BR cows (0.64% and 0.55%) than in SIN cows (0.11% and 0.13%).

Culling incidence was slightly lower in BR cows in parity 2 (1.13% vs. 1.21% in SIN) but higher in parity 3 (0.87% vs. 0.77%). Overall, abortion represented the most relevant reproductive disorder associated with brucellosis.

### 3.4. Economic Losses

**Figure 1:**
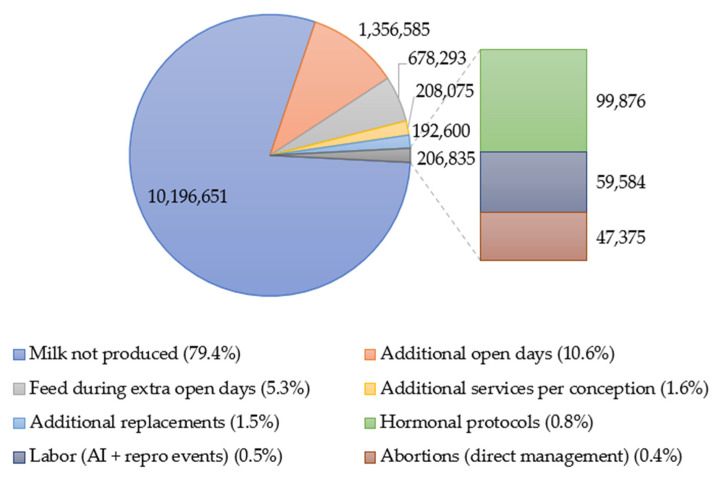
Three-dimensional pie chart illustrating the relative contribution of different cost components to the total estimated economic loss (US$ 12,839,039) associated with brucellosis-seropositive (BR) compared with seronegative (SIN) dairy cows. Milk not produced accounted for 79.4% (US$ 10,196,651) of the total loss, followed by additional open days (10.6%; US$ 1,356,585) and feed during extra open days (5.3%; US$ 678,293). Additional services per conception, additional replacements, hormonal protocols, labor associated with artificial insemination and other reproductive events, and direct abortion management contributed 1.6% (US$ 208,075), 1.5% (US$ 192,600), 0.8% (US$ 99,876), 0.5% (US$ 59,584), and 0.4% (US$ 47,375) of the total loss, respectively. Unit costs and data sources are described in the Materials and Methods section.

Figure 1 summarizes the estimated economic losses, showing that unproduced milk and additional DOs represented the largest cost components. The base milk price was USD 0.48/L [29,30]. BR cows exhibited an excess of +10 to +34 DOs compared with SIN cows; assuming a cost per DOs of USD 2.6–5.5/d [22,28], this corresponded to 271,317 extra days and an estimated loss of USD 1,356,585. Feeding costs during the additional DOs contributed USD 678,293 [27].

BR cows required approximately +0.4 to +1.0 additional SPCs in most parities (+8323 services in total), corresponding to USD 208,075 at an estimated USD 25/service [27,28]. Hormonal synchronization protocols (e.g., Ovsynch, USD 8–15/cow) added USD 99,876 [26,28]. Labor associated with AI and reproductive management events added approximately USD 47,375, assuming USD 250/event [24,25].

The incidence of culling was higher among BR cows (3.79%; 588/15,507) than among SIN cows (1.72%; 287/16,671), with an RD = 2.07 percentage points (95% CI: 1.71–2.43). This represents ~321 excess replacements, valuated at USD 600 net per cow, for an additional loss of USD 192,600 [7,8,21].

Sensitivity analyses confirmed the robustness of the economic estimates. When varying milk price (USD 0.40–0.55/L), cost per open day (US$2.6–5.5/d), and net replacement cost (USF 500–700/cow), total losses ranged between USD 11.9 and 13.7 M, with milk not produced remaining the dominant component (>75% of total loss).

Figure 1 illustrates the relative contribution of different cost components to the total estimated economic loss (USD 12,839,039) associated with brucellosis-seropositive (BR) compared with seronegative (SIN) dairy cows. Milk not produced accounted for 79.4% (USD 10,196,651) of the total loss, followed by additional open days (10.6%; USD 1,356,585) and feed during extra open days (5.3%; USD 678,293). The remaining components (additional services per conception, abortions, hormonal protocols, labor, and additional replacements) each represented ≤1.6% of the total loss. Unit costs and data sources are described in the Materials and Methods section.

## 4. Discussion

Mexico remains an endemic country for brucellosis in domestic animals; the National Campaign (NOM-041-ZOO-1995) establishes quarantine, vaccination, and culling as core strategies, but implementation remains limited due to producers’ reluctance and dependence on governmental incentives [12,13,16,32]. Dairy herds in Mexico exhibit a non-negligible prevalence (0.04–5.68% depending on the region) [33], and industrial pasteurization has allowed a gradual rather than immediate reduction of infected animals [34].

In geographically isolated settings (e.g., Terceira Island, Azores), eradication was achieved through mass RB51 vaccination combined with strict test-and-slaughter policies; however, replicating such success is difficult in densely populated and highly interconnected Mexican dairy regions characterized by heterogeneous infrastructure and limited financial resources [12,33,35].

In the present study, the management strategy for the BR group (gradual culling while pasteurizing milk) aimed to maintain short-term cash flow; however, vaccination with RB51 alone is insufficient under endemic conditions unless all positives are removed at the time of vaccination and newly positive animals are continuously culled over extended periods [36]. Although eradication has not yet been achieved in BR barns, the approach has allowed operational control of infection, whereas the SIN herd represents a longer-term success case.

The results confirm substantial productive and reproductive penalties associated with brucellosis. Across five parities, BR cows produced 3.5–5.0 L/cow/d less milk than SIN cows, consistent with findings from control programs where reductions in brucellosis incidence led to sustained increases in individual and herd-level milk yield [37]. Milk production shortfalls accounted for ~80% of the total estimated economic loss, aligning with economic frameworks indicating that milk income is the primary determinant of profitability in intensive dairy systems [22,27].

BR cows exhibited an excess of +10–34 DOs and higher SPCs, in agreement with previous evidence showing that improved reproductive performance enhances profitability by increasing milk output, calving frequency, and reducing reproductive and replacement costs [22,28]. The longer DIMs observed in BR cows (+14–27 d) provides no economic advantage, as this coincided with lower daily milk yield and an increased risk of health or reproductive disorders, supporting previous analyses indicating that lactation efficiency (rather than duration per se) is the key driver of profitability [27].

Abortion incidence was consistently higher in BR cows (up to 2.8%) than in SIN cows (0.7–1.6%), which aligns with national reports identifying abortion and infertility as leading causes of culling and with historical estimates of regional economic losses due to brucellosis [38,39]. Classic studies highlight critical windows for pregnancy loss, with expected late-gestation losses of ≈1–3%; our restriction to late-gestation data revealed BR rates at or above this upper threshold, whereas SIN cows remained below it, consistent with the known pathophysiology of brucellosis [40,41].

In a recent economic metamodel, abortions in new lactations (NLA) and re-inseminations following abortion (RA) were shown to reduce milk yield 19.4% and 7.3%, respectively. Moreover, RA events extended DOs by an average of 132 d and increased the culling risk by 1.9×, reinforcing the substantial productive and economic burden of abortion-related reproductive failures in dairy herds [25].

No significant differences were observed in age at conception or age at calving for heifers, suggesting that adverse effects of brucellosis manifest primarily in the post-partum period through abortions, prolonged DOs, and increased SPCs, contrasting with reports from smallholder tropical systems, where reproductive impairments tend to occur earlier in life [42].

Financially, total estimated losses (~USD 12.84 M) were primarily driven by milk shortfalls and extended DOs, which is consistent with standard valuations of DOs (USD 2.6–5.5/d) and pregnancy loss costs of approximately USD 897, a figure that closely corresponds to our estimated value of USD 828 [22,24]. Structured reproductive programs (e.g., Double-Ovsynch with resynchronization) can enhance annual net returns despite higher hormonal and labor expenses, owing to improved pregnancy rates and increased milk production [25,28,43]. The higher culling risk observed in BR cows (RD = 2.07 pp) translated into ~321 excess replacements; to avoid double counting across cost components, replacements were valued at their net cost (rearing cost minus salvage value), consistent with established approaches to longevity and replacement economics [7,8]. In production systems or markets with higher heifer prices or lower salvage values, this proportional contribution of this component to total losses could increase.

Comparable studies in Latin American semi-intensive systems (e.g., Brazil, Colombia, and Ecuador) have also reported milk yield reductions of 20–25% and prolonged calving intervals of 30–45 days among herds infected with Brucella abortus [5,44]. These findings confirm that economic losses remain substantial even in lower-input production systems. Likewise, Ref. [44] documented significant economic impacts and reproductive inefficiencies in semi-intensive Colombian dairies, while Ref. [45] summarized regional evidence of persistent seroprevalence and the suboptimal control of bovine brucellosis across Latin America.

Beyond production losses, maintaining seropositive animals for short-term economic reasons poses a public health concern, particularly in areas where unpasteurized milk is still traded or consumed. Therefore, eradication strategies should integrate economic, epidemiological, and biosafety perspectives, in alignment with One Health principles, to achieve both sustainable dairy productivity and effective zoonotic risk reduction.

The large sample size and parity stratification represent key strengths of this study; however, future research should incorporate stochastic modeling to account for interrelated cost components, assess the economics of biosecurity and targeted culling under local market conditions, and compare vaccination scenarios (RB51 vs. DIVA Concept) from both production–economic and biosafety perspectives.

## 5. Conclusions

In endemic settings, brucellosis significantly reduces milk yield and reproductive efficiency, with milk production losses representing the dominant economic component and extended DOs and increased SPCs acting as important secondary drivers. Progressive sanity control, strict biosecurity, strategic culling of positive animals, and structured reproductive management offer the most effective benefit–cost strategy to recover milk yield, shorten DOs, and reduce the abortion rate, thereby moving closer to the eradication of brucellosis.

## Figures and Tables

**Table 1:** Productive and reproductive parameters in (BR vs. SIN) cows across five parities.

Parity	Group	*n*	Milk Yield (L/d)	Days Open (d)	Services per Conception	Days in Milk (d)	SEM
1ra	BR	2617	29.6 ^a^	155 ^a^	3.5 ^a^	326 ^a^	±0.0852
	SIN	6414	34.4 ^b^	121 ^b^	2.5 ^b^	308 ^b^	
2da	BR	4705	32.4 ^a^	139 ^a^	3.5 ^a^	333 ^a^	±0.1963
	SIN	4473	36.3 ^b^	122 ^b^	3.0 ^b^	302 ^b^	
3ra	BR	4725	32.4 ^a^	135 ^a^	3.4 ^a^	320 ^a^	±0.1341
	SIN	3782	36.4 ^b^	125 ^b^	3.0 ^b^	303 ^b^	
4ta	BR	2926	32.4 ^a^	136 ^a^	3.3 ^a^	327 ^a^	±0.182
	SIN	1562	36.9 ^b^	121 ^b^	2.8 ^b^	308 ^b^	
5ta	BR	534	34.4 ^a^	126 ^a^	2.4 ^a^	319 ^a^	±0.4056
	SIN	440	38.4 ^b^	105 ^b^	2.5 ^a^	302 ^b^	

Different superscript letters within a column indicate significant differences between groups (*p* < 0.05). BR = cows seropositive for brucellosis; SIN = cows without brucellosis; SEM = standard error of the mean.

**Table 2:** Age at conception and calving in heifers (BR vs. SIN).

Group	*n*	Age at Conception (Months ± SEM)	Age at Calving (Months ± SEM)
BR	731	14.7 ± 0.09	23.7 ± 0.11
SIN	785	14.5 ± 0.09	23.7 ± 0.11

No significant differences were detected between groups (*p* ˃ 0.05). BR = cows seropositive for brucellosis; SIN = cows without brucellosis; SEM = standard error of the mean.

**Table 3:** Incidence of reproductive disorders (% of cows) by health (BR vs. SIN) and parity (1–5).

Parity	Group	*n*	Abortion (% ± SEM)	Metritis (% ± SEM)	RP (% ± SEM)	Culling (% ± SEM)
1	BR	2617	2.79 ^a^ ± 0.32	0.61 ± 0.15	0.19 ± 0.09	9.59 ^a^ ± 0.58
	SIN	6414	0.94 ^b^ ± 0.12	0.30 ± 0.07	0.03 ± 0.02	2.31 ^b^ ± 0.18
2	BR	4705	2.42 ^a^ ± 0.22	0.51 ^a^ ± 0.10	0.64 ^a^ ± 0.12	1.13 ± 0.15
	SIN	4473	0.89 ^b^ ± 0.14	0.04 ^b^ ± 0.03	0.11 ^b^ ± 0.05	1.21 ± 0.16
3	BR	4725	1.95 ^a^ ± 0.20	0.47 ^a^ ± 0.10	0.55 ^a^ ± 0.11	0.87 ± 0.14
	SIN	3781	0.69 ^b^ ± 0.13	0.19 ^b^ ± 0.07	0.13 ^b^ ± 0.06	0.77 ± 0.14
4	BR	2926	1.74 ± 0.24	0.48 ± 0.13	0.14 ± 0.07	5.54 ^a^ ± 0.43
	SIN	1562	1.54 ± 0.31	0.51 ± 0.18	0.06 ± 0.06	2.43 ^b^ ± 0.39
5	BR	534	2.25 ^a^ ± 0.64	0.37 ± 0.26	0.19 ± 0.19	15.17 ^a^ ± 1.55
	SIN	440	1.59 ^b^ ± 0.60	0.45 ± 0.32	0 ± 0.00	4.09 ^b^ ± 0.94

Incidence was calculated as (number of cases/total cows) × 100. Different superscript letters within a column indicate significant differences between groups (*p* < 0.05). RP = retained placenta; BR = cows seropositive for brucellosis; SIN = cows without brucellosis; SEM = standard error of the mean.

## Data Availability

The datasets presented in this article are not readily available as the data were provided by the company under a confidentiality agreement and are not publicly accessible. Requests to access the datasets should be directed to the corresponding author.

## Abbreviations

The following abbreviations are used in this manuscript:

BR	Cow group with brucellosis
SIN	Cow group without brucellosis
DOs	Days open
SPCs	Services per conception
DIMs	Days in milk
RP	Retained placenta
NOM	Mexican official norm
TMR	Total mixed ration
NEL	Net energy for lactation
MP	Metabolizable protein
RDP/RUP	Rumen degradable protein/rumen undegradable protein
GnRH	Gonadotropin release hormone
PGF_2α_	Prostaglandin F_2_ alpha
AI	Artificial insemination
RD	Risk difference
CWT	Hundredweight
CI	Confidence interval
